# Regions enriched for DNA repeats in chromosomes of Macrostomum mirumnovem, a species with a recent Whole Genome Duplication

**DOI:** 10.18699/VJ20.657

**Published:** 2020-10

**Authors:** K.S. Zadesenets, N.B. Rubtsov

**Affiliations:** Institute of Cytology and Genetics of Siberian Branch of the Russian Academy of Sciences, Novosibirsk, Russia; Institute of Cytology and Genetics of Siberian Branch of the Russian Academy of Sciences, Novosibirsk, Russia

**Keywords:** metaphase chromosome microdissection, DNA probes, repetitive DNA, mobile element transposition, FISH, DNA amplification, B chromosomes, микродиссекция метафазных хромосом, ДНК-пробы, повторенные последовательности ДНК, транспозиция мобильных элементов, FISH, амплификация ДНК, В-хромосомы

## Abstract

The free-living flatworm Macrostomum mirumnovem is a neopolyploid species whose genome underwent a recent Whole Genome Duplication (WGD). In the result of chromosome fusions of the ancient haploid chromosome set, large metacentric chromosomes were formed. In addition to three pairs of small metacentrics, the current karyotype of M. mirumnovem contains two pairs of large metacentric chromosomes, MMI1 and MMI2. The generation of microdissected DNA libraries enriched for DNA repeats followed by DNA probe preparation and fluorescent in situ hybridization (FISH) were performed. The DNA probes obtained marked chromosome regions enriched for different DNA repeats in the M. mirumnovem chromosomes. The size and localization of these regions varied in different copies of large chromosomes. They varied even in homologous chromosomes, suggesting their divergence due to genome re-diploidization after a WGD. Besides the newly formed chromosome regions enriched for DNA repeats, B chromosomes were found in the karyotypes of the studied specimens of M. mirumnovem. These B chromosomes varied in size and morphology. FISH with microdissected DNA probes revealed that some Bs had a distinct DNA content. FISH could paint differently B chromosomes in different worms and even in the same sample. B chromosomes could carry a bright specific fluorescent signal or could show no fluorescent signal at all. In latter cases, the specific FISH signal could be absent even in the pericentromeric region of the B chromosome. Possible mechanisms of B chromosome formation and their further evolution are discussed. The results obtained indicate an important role that repetitive DNAs play in genome re-diploidization initiating a rapid differentiation of large chromosome copies. Taking together, karyotype peculiarities (a high level of intraspecific karyotypic diversity associated with chromosome number variation, structural chromosomal rearrangements, and the formation of new regions enriched for DNA repeats) and some phenotypic features of M. mirumnovem (small body size, short lifecycle, easy maintenance in the laboratory) make this species a perspective model in the studies of genomic and karyotypic evolution in species passed through a recent WGD event.

## Abstract (ru)

Свободноживущий плоский червь Macrostomum mirumnovem – неополиплоидный вид, его геном претерпел недавнюю полногеномную дупликацию (Whole Genome Duplication, WGD). В результате слияния гаплоидного хромосомного набора в его кариотипе произошло формирование двух новых крупных хромо- сом, MMI1 и MMI2. Создание микродиссекционных ДНК-библиотек, обогащенных повторенными последо- вательностями ДНК, и их последующая гибридизация in situ с метафазными хромосомами M. mirumnovem выявили в этих хромосомах районы, обогащенные повторенными последовательностями ДНК. Разные ДНК- пробы устанавливали в хромосомах M. mirumnovem районы, обогащенные разными повторенными после- довательностями. Локализация и размер этих районов варьировали в разных копиях крупных хромосом, это предполагало их дивергенцию и снижение уровня гомологии, что может после полной дупликации ге- нома приводить к его редиплоидизации. Помимо возникших de novo районов хромосом основного набора, обогащенных повторенными последовательностями, в кариотипе у большинства исследованных особей обнаружены В-хромосомы, которые варьировали по размеру и морфологии. Различия в составе ДНК у этих В-хромосом были показаны с помощью флуоресцентной гибридизации in situ (FISH) с полученными микро- диссекционными ДНК-пробами на хромосомном материале, взятом от разных животных. Флуоресцентная гибридизация in situ этих ДНК-проб по-разному окрашивала В-хромосомы, содержащиеся в кариотипах у раз- ных особей M. mirumnovem. Часть В-хромосом интенсивно окрашивалась при проведении FISH, тогда как на других В-хромосомах гибридизационных сигналов не было. Специфический FISH-сигнал отсутствовал даже в прицентромерных районах таких В-хромосом. В настоящей статье обсуждаются возможные механизмы возникновения и последующей эволюции В-хромосом у M. mirumnovem. Полученные результаты указывают на важную роль повторенных последовательностей, которую они могут играть в процессе реорганизации генома, приводя к быстрой дифференциации дуплицированных копий хромосом. Высокий уровень внутри- видового кариотипического разнообразия по численным и структурным хромосомным перестройкам и по формированию новых хромосомных районов, обогащенных повторенными последовательностями, а также небольшой размер тела (~2 мм) и простота поддержания лабораторных культур M. mirumnovem делают этот вид перспективной моделью в исследованиях геномной и кариотипической эволюции видов, недавно про- шедших полногеномную дупликацию.

## Introduction

Comparative genomics opened up new possibilities for studies of mechanisms of whole-genome duplication (WGD), as well as its consequences after the large-scale changes of genome involving almost all of the genes. The most of the existing species of plants and animals were resulted from at least one round of WGD, more often a few WGD events (Wendel, 2000; Panopoulou et al., 2003; Dehal, Boore, 2005). In different phylogenetic lineages, these WGDs took place hundreds of MYA. Hence, the genomes of modern species are paleoploids, and they contain only traces of WGDs (Dehal, Boore, 2005). Therefore, comparative analysis of their genomes can provide only limited knowledge about mechanisms of polyploid formation and further reorganization of the duplicated genome. There are two main mechanisms of WGD: autopolyploidy resulted in autotetraploid genome formation, and allopolyploidization, i. e. doubling of hybrid genome after interspecific hybridization. In the latter case, a new allopolyploid genome contains both parental genomes, and it can retain many traits of its hybrid ancestor. The key role of WGD in genome evolution in animals was considered in the recent review (Zadesenets, Rubtsov, 2018).

WGD events occurred in genomes of different phylogenetic lineages (yeasts, plants, animals). Many studies performed by different research groups have uncovered that the number of WGDs can distinguish for different phylogenetic lineages: for instance, in rotifer – 1 WGD, in the vast majority of vertebrates – 2, in many plants – 3, in salmonids – 4 (Dehal, Boore, 2005; Glasauer, Neuhauss, 2014; Kenny et al., 2018). The genome evolution of Brassica rapa contained alternating rounds of multiplication events, including both whole-genome duplication and even triplication (Moghe et al., 2014). Some researchers suggest that WGD creates conditions for following large evolutionary transformations, despite other scientists take a different view, suggesting post-WGD species are mostly evolutionary dead-end (Mayrose et al., 2011; Soltis et al., 2014). For many years the most of studies were devoted to the analysis of polyploidy and WGD in plants that was linked with the limitations of the available methodological possibilities. Development of new methods in molecular genetics, improvement of NGS technologies have fundamentally changed the WGD studies in animals (Zadesenets, Rubtsov, 2017b). In a result, comparative genomics has arisen, and more and more animal species continue to be involved in these studies (Comparative Genomics, 2000). The limited number of species underwent a recent WGD is a challenge in researches devoted to WGD and early-stage genome reorganization after a WGD in animals (Zadesenets, Rubtsov, 2018).

It should be noted that a large number of polyploid variants are likely to be evolutionary dead-ends (Mayrose et al., 2011; Barker et al., 2016). They represent new, but less competitive variants in comparison with their diploid ancestors. Nevertheless, traces of WGDs revealed in genomes of many existing species indicate to a significant contribution of WGD in the formation of new mechanisms of environmental adaptation (Glasauer, Neuhauss, 2014; Fisher et al., 2018). Although WGD can be considered as a key event in the formation of new phylogenetic lineages, the possibilities of studying a WGD and the processes it triggers in the followed stages of genome evolution are limited. The rapid progress in the development of new methods of molecular genetics and genomics has opened up new opportunities and perspectives in the evolutionary studies. However, the number of perspective model organisms for studying WGD remains extremely low. The involvement in studies of new species, whose genomes passed through a recent WGD, is one of the most urgent tasks. The group of species of free-living flatworms of the genus Macrostomum may turn out to be one of such promising model species.

Molecular cytogenetic analysis of the M. lignano chromosomes uncovered that the main traits of its genome and karyotype organization differ from that of the other Macrostomum species, and these peculiarities are linked with a recent WGD event in its genome evolution (Zadesenets et al., 2017a, c). The karyotypes of many Macrostomum species consist of a low number of small metacentric chromosomes (Egger, Ishida, 2005; Zadesenets et al., 2016; Schärer et al., 2020). It was suggested that a basal chromosome number was 2n = 6, but due to the WGD, the doubled chromosome number led to the increased karyotype 2n = 12 in some Macrostomum species. Chromosome fusions of all chromosomes of one haploid chromosome set of ancestor have resulted in the decreased chromosome number (from 12 to eight chromosomes) in the M. lignano karyotype (Egger, Ishida, 2005; Zadesenets et al., 2016). Moreover, paralogous regions revealed in the M. lignano chromosomes accumulated some differences that more likely arose after a WGD event (Zadesenets et al., 2017a, c). The uncovered karyotype instability in M. lignano is considered to be a consequence of the ongoing rediploidization processes in its genome. The karyotyping of new Macrostomum species led us to explore a new species, namely M. mirumnovem arose through a recent independent round of WGD. The genome evolution of this species accompanied by intensive chromosome reshuffling resulted in chromosome fusions, structural chromosome rearrangements, formation of new repeat-enriched regions, and B chromosome formation (Zadesenets et al., 2020). Based on the cytogenetic analysis of the M. mirumnovem chromosomes, we concluded that expansion and amplification of repetitive DNA play a significant role in the reorganization of its karyotype. By generation of microdissected DNA probes enriched with DNA repetitive sequences, we studied the distribution of these DNA repeats in the M. mirumnovem chromosomes.

## Material and methods

**Laboratory outbred culture of the free-living flatworm M. mirumnovem.** The laboratory outbred culture of M. mirumnovem, kindly provided by Professor Lukas Schärer (Zoological Institute of the University of Basel, Switzerland), was used in the current study. The culture of M. mirumnovem has been cultivated under the laboratory conditions for three years. The first karyotyping was performed three months after sampling the M. mirumnovem worms from natural populations (Zadesenets et al., 2020). Already at this stage, a high level of karyotypic diversity was revealed. The most common karyotype consisted of nine chromosomes: the largest unpaired metacentric (MMI1), a pair of large metacentrics (MMI2) a bit smaller in size than the MMI1, and three pairs of small metacentric chromosomes MMI3–MMI5 (Schärer et al., 2020; Zadesenets et al., 2020).

In the chromosomes thus formed, the active expansion of transposable elements (TEs) and amplification of DNA sequences probably led to the formation of numerous regions enriched for repetitive sequences (Zadesenets, Rubtsov, 2020). Single-worm karyotyping, carried out before microdissection of small metacentrics, showed an increase in the karyotypic diversity among the worms of the laboratory culture, as well as the formation of B chromosomes in it. In the current study, we used the metaphase plates with the 2n = 9 karyotype to generate the microdissected DNA probes. For fluorescence in situ hybridization (FISH) metaphase chromosome slides obtained from individual worms with different karyotype variants were used.

**Preparation of metaphase chromosome plates.** Regular karyotyping of individual worms of the laboratory culture of M. mirumnovem was done as was previously described (Zadesenets et al., 2016). We checked at least ten metaphase spreads per each specimen. For microdissection, we prepared chromosome slides using a cell suspension technique that provides a better preservation of DNA in metaphase chromosomes. To make a suspension of fixed mitotic cells, we used 100 mature worms, as was earlier described (Zadesenets et al., 2016). Metaphase plates were spread on a wet surface of cold coverslips (60 × 24 × 0.17 mm). Preparation of metaphase spreads on coverslips allowed us to use a maximum magnification of the inverted microscope AXIOVERT10 (ZEISS, Germany) during microdissection.

**Staining of metaphase chromosomes.** For routine karyotyping the metaphase chromosomes were stained with a fluorescent dye DAPI (4′,6-diamidino-2-phenylindole solution) dissolved in the VECTASHIELD® mounting medium containing antifade (Vector Laboratories Inc., Burlingame, CA, USA). Metaphase chromosome slides prepared for microdissection were stained in a 2 % Giemsa solution (in 1xPBS, pH = 7.2, PanEco, Moscow, Russia) (Zadesenets, Rubtsov, 2020).

**Microscopic analysis** of chromosome preparations was performed at the Center for the collective use of microscopic analysis of biological objects of the SB RAS at the Institute of Cytology and Genetics SB RAS (Novosibirsk, Russia) using an Axioplan 2 luminescent microscope (ZEISS) equipped with a CCD camera and a set of filters # 49, # 10 and # 15 (ZEISS). Registration and subsequent processing of microscopic images of chromosomes was performed using the ISIS4 software (METASystems GmbH, Germany).

**Microdissection of metaphase chromosomes and generation of microdissected DNA probes.** Microdissection of metaphase chromosomes was used for the generation of the DNA probes from the M. mirumnovem chromosomes as was described earlier (Zadesenets et al., 2016). To amplify the DNA of the dissected chromosomal material, we used a standard variant of sequence-independent polymerase chain reaction (PCR) (Zadesenets et al., 2017a, c). The procedure of microdissection from the selection of the metaphase plate to the transfer of dissected material into a PCR tube with a reaction mixture were described in detail (Zadesenets, Rubtsov, 2020). In the current study, we collected two single copies of small chromosomes.

The difference in the preparation of DNA probes from the standard protocol consisted in modifying the composition of the collection buffer, into which the dissected chromosome material was directly collected (~40 nl in the drawn-out silicone tip of the Pasteur pipette). The collection drop contained a proteinase and DNA fragmentation buffer (commercial Whole Genome Amplification 4 (WGA4) kit, Sigma-Aldrich, USA) and 0.1 % non-ionic detergent Triton X-100 (VWR Life Science AMRESCO, USA). All subsequent stages of DNA preparation for amplification and directly DNA amplification were carried out according to the previously described protocol (Zadesenets et al., 2016, 2017a, c) with an increased number of PCR cycles (up to 35 cycles) at the last stage of the preparation of a microdissected DNA library.

Generation of microdissected DNA libraries involves two steps: (1) the generation of DNA fragments (from the dissected chromosomal material) flanked by the corresponding sequences (WGA4 kit, Sigma-Aldrich, USA) and (2) the amplification of such DNA fragments in PCR. Depending on the efficiency of obtaining the flanked DNA fragments in the first stage, there are two different variants for the DNA content of the obtained DNA libraries. In the first one, the sequences repeated many times in the genome and a significant part of the unique and low repetitive sequences are involved in amplification. In the second one, the part of genomic DNA sequences involved in amplification was reduced and mostly highly repeated sequences appeared to be amplified. The representation of unique DNA sequences in the obtained microdissected DNA libraries is significantly reduced with a decrease in the efficiency of generation of DNA fragments capable of amplification at the first stage. In a result, the generated DNA libraries are enriched with repetitive sequences.

An increase in the number of PCR cycles at the second stage of generation of microdissected DNA library allows to obtain the required amount of DNA product. The modification of the collection buffer used in the first stage of generation DNA libraries led to the production of DNA libraries that were enriched in highly repeated sequences. The presence mostly of highly repetitive DNA in the obtained DNA libraries was confirmed by the complete absence of a signal in most euchromatin regions of chromosomes after FISH with DNA probes obtained on their basis.

The resulting PCR product was labelled in 20 additional PCR cycles in the presence of fluorochrome-conjugated nucleotides: Flu-12-dUTP [fluorescein-5(6)-carboxamidocaproyl-[ 5(3-aminoallyl)2′-deoxyuridine-5′-triphosphate] (Biosan, Novosibirsk, RF) or TAMRA-5-dUTP [tetramethylrhodamine- 5(6)-(5-[3-carboxamidoallyl]-2′-deoxyuridine 5′-triphosphate)] (Biosan) (Zadesenets et al., 2017a, c). GenomePlex® Whole Genome Amplification Reamplification kit (WGA3) (Sigma-Aldrich, USA) was used for labelling.

**Fluorescent in situ hybridization** on metaphase chromosomes of DNA probes with metaphase chromosomes of M. mirumnovem was performed according to the previously described protocol (Zadesenets et al., 2017a, c), without suppression of the repetitive DNA hybridization. During FISH, chromosome slides prepared from individual worms of M. mirumnovem were analyzed. When FISH was carried out with separate microdissected DNA probes (S3 or S4), as well as when two-colour FISH with DNA probes S3 and S4, was performed ten or more specimens of M. mirumnovem were studied.

## Results and discussion

**Fig. 1.**
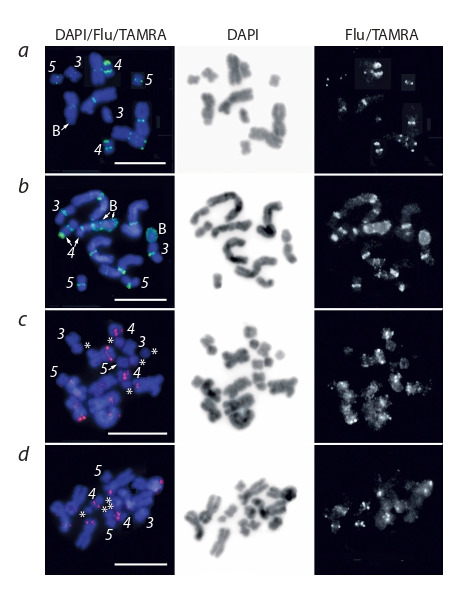
Fluorescent in situ hybridization of the microdissected DNA probes S3 (red signal) and S4 (green signal) with metaphase chromosomes obtained from different specimens of M. mirumnovem. The chromosomes differ in the size of the regions enriched for repetitive DNA sequences (а–d ). The inverted image of DAPI-staining and separate channels for fluorochromes Flu/TAMRA are presented. The chromosomes MMI3–MMI5 are indicated by numbers (3–5, correspondently) and В chromosome(s) is/are marked by the letter “B”. The suggested B chromosomes are shown with asterisks (small chromosomes having the size and morphology distinct from that of the chromosomes MMI3–MMI5). Scale bar 10 μm.

**Fig. 2.**
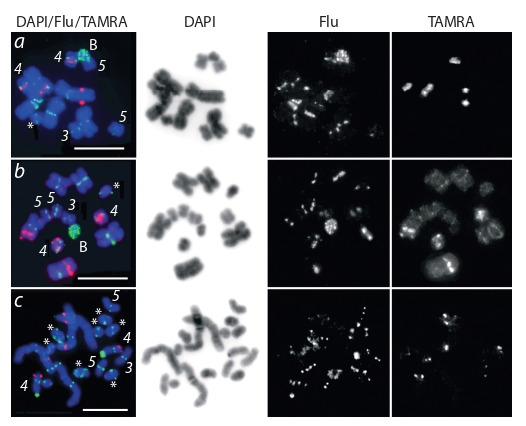
Two-colour fluorescent in situ hybridization of the microdissected DNA probes S3 (red signal) and S4 (green signal) with metaphase chromosomes of high (a–b) and low (c) condensation level. Metaphase spreads were obtained from various samples of M. mirumnovem. The chromosomes differ in the size of the regions enriched in repetitive DNA sequences (а–c). The inverted image of DAPI-staining and separate channels for fluorochromes Flu/TAMRA are presented. For trait designations see Fig. 1.

**Generation of DNA probes from chromosomes of M. mirumnovem and FISH of the obtained DNA probes with metaphase chromosomes.** The S3 and S4 DNA probes were obtained from single copies of two small metacentrics. FISH with the DNA probes on metaphase chromosomes of M. mirumnovem revealed chromosome regions enriched for repetitive sequences homologous to DNA in the DNA probes S3 and S4 (Fig. 1, 2). The regions painted by the DNA probes S3 or S4 will be called S3D- or S4D-regions, respectively, i. e. the regions detected by FISH using the DNA probes S3 and S4. In our study, the definition of precise localization of these regions in metaphase chromosomes of M. mirumnovem posed a certain problem of the description based on their morphology and differential staining.

Fluorescent in situ hybridization with the S4 DNA probe on the large metacentric chromosomes, MMI1 and MMI2, gave bright fluorescence exactly in the region of their primary constriction, and additional signals in some regions of their chromosome arms (see Fig. 1, 2). In addition to FISH signals in large chromosomes, the DNA probe S4 gave an intense signal in the pericentromeric region of the chromosome MMI4. In some specimens, the distal region of q-arm of MMI4 was also intensively painted (see Fig. 1, a, b, 2, c). The weak signals (on the edge of being able to detect them) were also detected in the pericentromeric regions of the chromosomes MMI3 and MMI5.

Due to the small block of the pericentromeric structural heterochromatin in the M. mirumnovem chromosomes, it is hardly possible to conclude whether the S4D-regions were located directly in the pericentromeric regions or in adjacent euchromatin regions even in the large chromosomes MMI1 and MMI2. Localization of FISH signal on the condensed metaphase chromosomes MMI3–MMI5 often turned out to be even more complicated (see Fig. 1, a, b). However, it should be noted that on less condensed chromosomes MMI3– MMI5 with a pronounced primary constriction, the FISH signal of the DNA probe S4 was always localized in this region (see Fig. 2, c).

Thus, taking into account the small size of the pericentromeric heterochromatic regions, the question whether the S4Dregions are located in the pericentromeric heterochromatin of the chromosomes or the proximal euchromatin of p- or q-arms, remains open. For simplicity, a further description of the obtained results, we will define these regions pericentromeric, recognizing some incorrectness of the use of this term.

Given the specificity in the description of FISH results, we can conclude that the DNA probe S4 painted the pericentromeric regions of all A-chromosomes. The intensity of FISH signals in different chromosomes varied significantly, changing from the intense signal in the region of the primary constriction of some copies of chromosomes MMI1, MMI2, and MMI4 to the weak separate signals on both chromatids of small metacentrics MMI3 and MMI5 (see Fig. 1, a, b). The variation in fluorescence intensity of the signal of the DNA probe S4 in regions of different chromosomes, including different copies of homologous chromosomes, was probably derived from the different copy number of the corresponding repeats in these regions. However, it cannot be ruled out that in some cases such differences may be based on the differences in homology level between repetitive sequences in these regions and the DNA of the S4 probe. It should be noted that additionally to specific signals in the regions of A-chromosomes, the DNA probe S4 weakly painted some B-chromosomes (see Fig. 1, b, 2, a, b).

Intense FISH signals of the DNA probe S3 localized only in small chromosome MMI4 and in large metacentric chromosomes (MMI1 and MMI2). Furthermore, two-colour FISH with the DNA probes S3 and S4 showed that the large chromosomes are enriched for different highly repeated sequences. Moreover, the revealed S3D- and S4D-regions were not colocalized (see Fig. 2, a–c). Even in the proximal region of MMI4, where the S3D- and S4D-regions were localized close to each other, no co-localized FISH signals of the DNA probes S3 and S4 were observed.

In one specimen, a transition of the S3D-region to the distal region of the p-arm of MMI4 was revealed, which is visible on low condensed chromosomes (see Fig. 2, c). It is likely that this transition could be resulted from an inversion involving one of the breakpoints located between the S3D and S4D regions. The obtained FISH results indicated that different repetitive sequences belonging to MMI4 were involved in DNA amplification during generation of the DNA probes S3 and S4.

The low-intense FISH signals of the DNA probe S3 (on the edge of the possibility of their detection) were revealed in the regions of small chromosomes MMI3 and MMI5. Either the revealed regions probably contained a relatively low copy number of DNA repeats homologous to that of the DNA probe S3, or DNA repeats in them had a low homology level with the DNA of the probe S3. We should note that the DNA probe S3 intensively painted the regions in the arms of some copies of large metacentrics (see Fig. 1, a–d, 2, a–c). Some of these regions were painted even more intensively than the proximal part of MMI4.

Summarizing data on FISH with the DNA probes S3 and S4 it is possible to state that two different types of large chromosomes were revealed in the karyotypes of studied specimens of M. mirumnovem. One of them was characterized by the presence of strong hybridization signals in their arms, while another showed the level of specific FISH signal intensity comparable to registered ones in the regions of small chromosomes MMI3 and MMI5 (see Fig. 1, 2).

**Possible mechanisms for the formation of regions enriched for repetitive sequences and their following evolution.** The question of the molecular mechanisms providing the variability of the regions enriched for DNA repeats in size and location remains open. However, it is necessary to take into account that chromosomes MMI1 and MMI2 contain extended regions homologous to the euchromatic regions of MMI4 (Zadesenets et al., 2020). Besides variable S3D- and S4D-regions (in terms of localization and size) observed in the MMI1 and MMI2, the S3D- and S4D-regions were also detected in the chromosome MMI4, but only their traces were found in the MMI3 and MMI5. Probably, the fusions of ancestral chromosomes that formed the large metacentrics were accompanied by amplification of DNA repeats derived from MMI4. Further expansion and amplification of repetitive DNA sequences could lead to the divergence of the formed large metacentrics. The distribution of such regions within the chromosome could also occur as a result of inversions, as it was shown in case of transition of the S3D-region in the chromosome MMI4 in one of the analyzed specimens of M. mirumnovem (see Fig. 2, c).

Based on the results obtained in our study, it is not possible to estimate what kind of repetitive sequences are present in the microdissected DNA probes. In future, the answer to this question can be obtained by cloning DNA fragments from the generated DNA libraries and subsequent FISH with DNA probes prepared from these DNA fragments. Previously, this approach was successfully applied to analyze the DNA composition of the B chromosome of the locust species, Podisma kanoi (Bugrov et al., 2007). An alternative way for studying the DNA composition of the microdissected DNA libraries is their NGS sequencing. Earlier, this approach was applied to determine the DNA composition of Bs of the Korean field mouse Apodemus peninsulae from East Asia, the yellow-necked mouse Apodemus f lavicolis, and a small supernumerary marker chromosome in humans (Makunin et al., 2018). However, the latter experimental design implies following verification of the results of NGS sequencing using DNA probes generated based on the obtained sequences, since a certain amount of DNA always contaminate microdissected DNA libraries. Additionally, for a successful and efficient interpretation of the data derived from the microdissected library sequencing, it is desirable to have a reference genome assembly or at least genome draft of the studied species (Zadesenets et al., 2017b). In the case of M. mirumnovem, it is problematic due to the prominent karyotype and genome instability, and therefore it requires consideration of alternative approaches.

**B chromosomes of M. mirumnovem.** In addition to the regions of A chromosomes, the DNA probe S4 gave specific signal in some Bs (see Fig. 1, b, 2, a–c). These results allow us to propose one of the scenarios for the formation of such Bs. They could be originated from the pericentromeric region of one of the A chromosomes as a result of chromosome breaks, leading to the arising of a small additional chromosome. At the beginning, this chromosome (or proto-B chromosome) consisted of the pericentromeric region of the ancestral A chromosome, and the subsequent amplification of repetitive DNA sequences homologous to DNA of the microdissected DNA probes and other different DNA repeats. The uneven and not intense specific fluorescence from the DNA probe S4 on the B indicates the presence of such different DNA repeats in the B chromosome. It is worth noting that these DNA repeats can derived both from the pericentromeric region of the ancestral A chromosome and from different chromosome regions. For instance, some Bs gave no specific signal after FISH with the DNA probe S4. Moreover, specific FISH signal was absent even in the pericentromeric regions of such B chromosomes (see Fig. 1, a), which indicates the amplification of different types of DNA repeats during the formation of the Bs, and, probably, their different origin.

## Conclusion

Using microdissected DNA probes generated by the modified protocol and containing predominantly highly repeated DNA sequences, we identified the regions enriched in repetitive DNA sequences in the M. mirumnovem chromosomes. We have shown that enrichment of such chromosomal regions for DNA repeats could vary substantially. The formation and following changes of these regions in large metacentrics MMI1 and MMI2 could lead to differentiation of the copies of homologous chromosomes. Additionally, the obtained results indicate that the DNA content in different copies of B chromosomes could differ among individuals of M. mirumnovem. The repetitive DNA sequences homologous to DNA repeats from the proximal regions of A chromosomes took part in the formation of at least some B chromosomes.

## Conflict of interest

The authors declare no conflict of interest.
